# Supplementary material for: Group-based local adaptive deep multiple kernel learning with lp norm
Source: PLoS One. 2020 Sep 17;15(9):e0238535. doi: 10.1371/journal.pone.0238535 (PMC7498035; doi:10.1371/journal.pone.0238535)
Supplement: Attachment — Submitted filename: renamed_f587d.docx [file pone.0238535.s001.docx]

Dear Reviewers，

     Thank you for your review of our manuscript (ID: PONE-D-20-09329), which is titled “**Group-based local adaptive deep multiple kernel learning with lp norm**”. We appreciate your concerns and suggestions, and have revised our manuscript accordingly.

The followings are our responses for your comments.

Point1:

The manuscript must describe a technically sound piece of scientific research with data that supports the conclusions. Experiments must have been conducted rigorously, with appropriate controls, replication, and sample sizes. The conclusions must be drawn appropriately based on the data presented.

**Response1:**

**We supplement and refine the experimental data, add corresponding analysis and further explain the ambiguities. All the modifications are marked in Yellow in the paper.**

Point2:

Has the statistical analysis been performed appropriately and rigorously?

**Response2:**

**Thanks to the reviewers for affirming that there are detailed statistical analysis in my paper. In the experiment of UCI datasets, we have discussed comparison of classification performance, the effect of group numbers on classification performance and the effect of layer numbers on classification performance. In the experiment of Caltech-256 datasets, we randomly select five types of data with similar shapes: bowling-ball, car-tire, desk-globe, roulette-wheel and sunflower-101. When these five datasets are respectively used as positive, we have analyzed the classification accuracy under different numbers of layers and groups. Special thanks for your good suggestions.**

Point3:

The PLOS Data policy requires authors to make all data underlying the findings described in their manuscript fully available without restriction, with rare exception (please refer to the Data Availability Statement in the manuscript PDF file). The data should be provided as part of the manuscript or its supporting information, or deposited to a public repository. For example, in addition to summary statistics, the data points behind means, medians and variance measures should be available. If there are restrictions on publicly sharing data—e.g. participant privacy or use of data from a third party—those must be specified.

**Response3:**

**The data in my experiment has been provided as part of the paper.** There are no restrictions on publicly sharing data. The url is: https://github.com/fage8/GLDMKL.git.

Point4:

PLOS ONE does not copyedit accepted manuscripts, so the language in submitted articles must be clear, correct, and unambiguous. Any typographical or grammatical errors should be corrected at revision, so please note any specific errors here.

**Response4:**

**The language usage, spelling and grammar have been completely revised in the paper. All the modifications are marked in Yellow in the paper. For example, lp norm is modified to the lp norm. classical DMKL is modified to the classical DMKL.**

More reasons and modifications have been noted in the article, all the modifications are marked in Yellow in the paper. Special thanks to you for your good comments.

Sincerely yours,

Shengbing Ren, Fa Liu, Weijia Zhou, Xian Feng and Chaudry Naeem Siddique
